# Exploring the impact of ketogenic diet on multiple sclerosis: obesity, anxiety, depression, and the glutamate system

**DOI:** 10.3389/fnut.2023.1227431

**Published:** 2023-08-25

**Authors:** Jose Enrique de la Rubia Ortí, María Cuerda-Ballester, Claudia Emmanuela Sanchis-Sanchis, Jose María Lajara Romance, Esther Navarro-Illana, María Pilar García Pardo

**Affiliations:** ^1^Department of Nursing, Catholic University of Valencia San Vicente Mártir, Valencia, Spain; ^2^Doctoral School, Catholic University of Valencia San Vicente Mártir, Valencia, Spain; ^3^Faculty of Legal, Economic and Social Sciences, Catholic University of Valencia San Vicente Mártir, Valencia, Spain; ^4^Department of Psychology and Sociology, University of Zaragoza, Teruel, Spain

**Keywords:** multiple sclerosis, ketogenic diets, obesity, anxiety, depression, glutamate

## Abstract

**Background:**

Multiple sclerosis (MS) is a neurodegenerative disorder. Individuals with MS frequently present symptoms such as functional disability, obesity, and anxiety and depression. Axonal demyelination can be observed and implies alterations in mitochondrial activity and increased inflammation associated with disruptions in glutamate neurotransmitter activity. In this context, the ketogenic diet (KD), which promotes the production of ketone bodies in the blood [mainly β-hydroxybutyrate (βHB)], is a non-pharmacological therapeutic alternative that has shown promising results in peripheral obesity reduction and central inflammation reduction. However, the association of this type of diet with emotional symptoms through the modulation of glutamate activity in MS individuals remains unknown.

**Aim:**

To provide an update on the topic and discuss the potential impact of KD on anxiety and depression through the modulation of glutamate activity in subjects with MS.

**Discussion:**

The main findings suggest that the KD, as a source of ketone bodies in the blood, improves glutamate activity by reducing obesity, which is associated with insulin resistance and dyslipidemia, promoting central inflammation (particularly through an increase in interleukins IL-1β, IL-6, and IL-17). This improvement would imply a decrease in extrasynaptic glutamate activity, which has been linked to functional disability and the presence of emotional disorders such as anxiety and depression.

## Introduction

Multiple sclerosis (MS) is a neurodegenerative disease characterized by multifocal and temporally dispersed damage to the central nervous system (CNS) (1, 2), affecting approximately 2.3 million people worldwide, with an increasing incidence (3). Currently, there is no specific treatment to cure the disease and the disease-modifying treatments administered aim to improve symptoms and slow down disease progression (4).

Prominent among the clinical presentations are fatigue and progressive muscular atrophy (5) resulting in functional disability (6, 7), along with notable cognitive and behavioral issues such as anxiety and depression (8).

At the central level, three pathogenic mechanisms are involved: excessive buildup of intra-axonal Ca^2+^ (9), axonal demyelination causing degeneration due to the lack of trophic support provided by myelin (10), and an inflammatory process triggered by immune system alterations mediated by reactive T and B lymphocytes (11). These mechanisms ultimately disrupt mitochondrial activity, resulting in decreased ATP production and increased oxidative stress. Demyelination and neurodegenerative changes are more commonly observed in deep gray matter, which directly impacts atrophy and clinical deterioration (12, 13).

Functional disability emerges as a prominent clinical factor directly associated with gray matter atrophy (14). This disability is quantified using the Expanded Disability Status Scale (EDSS), which represents a reliable tool for assessing life expectancy (15). Furthermore, it has been established that functional impairment, as assessed by the EDSS, directly influences emotional well-being of, particularly in relation to the presence of depression and anxiety (16–18). Therefore, it appears relevant to identify the factors that contribute to disability and their correlation with different variables, with obesity emerging as notably significant.

Most MS individuals are obese, and greater levels of obesity correlate with increased disability (19–21). Excessive adiposity has been linked not only to insulin resistance but also to an altered profile of various lipoproteins in the bloodstream (22, 23). It should be emphasized that these metabolic changes resulting from hyperinsulinemia may be responsible for the inflammatory process (24).

Inflammation is a prevalent feature in all MS subjects, prompting endeavors to identify inflammatory mediators as potential diagnostic biomarkers (25). While numerous markers have been discovered, certain proinflammatory interleukins hold particular significance in the inflammatory process. Specifically, elevated levels of IL-17, IL-6, and IL-1β have been highlighted by several authors (26). The rise in IL-17 levels in both the serum and cerebrospinal fluid (CSF) correlates with disease activity (27, 28). Furthermore, the synthesis of IL-17 is less sensitive to hydrocortisone inhibition compared to IL-6, and its levels are more significantly associated with active brain lesions in MS (29). The relationship between IL-6 and IL-17 is also interesting, as IL-6 is involved in the induction of IL-17-producing T cells, even during the remission phase. This leads to increased levels of IL-17, which positively correlate with disease severity according to the EDSS. This highlights the particular relevance of IL-6 in the pathogenesis of MS (30). In fact, elevated levels of IL-6 interfere with synaptic plasticity mechanisms, thereby affecting the ability to compensate for the clinical manifestation of new brain lesions in individuals with relapsing–remitting multiple sclerosis (RRMS) (31). Furthermore, IL-6 has been identified as an important mediator in the association between body mass index (BMI), adiposity, and risk of MS (32). Regarding IL-1β, although there are many aspects that require further investigation, it appears to be clearly related to the pathogenesis of the disease, as it acts on subsets of human TH cells expressing the IL-1R receptor (33).

## Inflammation in MS and anxiety and depression

Considering the significance of inflammation in the development of mental disorders, a direct association between elevated levels of inflammatory cytokines in the bloodstream and the presence of depressive and anxiety symptoms (34–37) has been established by numerous studies. Among these pro-inflammatory interleukins, IL-6, IL-1β, and IL-17 are particularly relevant in their association with emotional disturbances (38). According to Lee STH (39), IL-6 is associated with depression as a predictive factor, on average 6 years prior to its onset, and is positively correlated with its increase (40, 41), as well as the presence of anxiety symptoms (42, 43). The link between interleukin and these symptoms has been attributed to oxidative stress triggered by elevated levels of IL-6, which directly influence the normal functioning of the brain (44, 45), through hyperstimulation of the hypothalamic–pituitary–adrenal (HPA) axis (46). This oxidative stress and increased depression lead to demyelination and cerebral atrophy, accompanied by elevated concentrations of inflammatory mediators such as tumor necrosis factor (TNFα) and specifically interleukin IL-6, along with IL-1α and IL-1β, in the serum and CSF (47). Among the two IL-1 s, however, the role of IL-1β appears to be more relevant, particularly in relation to anxiety and depression. In fact, improvement of these symptoms following pharmacological treatment has been directly associated with a significant decrease in this interleukin (48), identifying it as a therapeutic target for the treatment of depression in individuals with MS. Finally, regarding IL-17, its elevated levels have recently been associated, along with IL-6, with subjects with first-episode depressive disorder (FDD) (49), and it also stands out among the various interleukins as being most strongly associated with the presence of anxiety, being considered as a severity indicator (50). Furthermore, it is interesting to highlight that in autoimmune diseases such as psoriasis, it may be a link between immune dysregulation, the predominant obesity in the condition and depression (51). In this regard, it is worth recalling the autoimmune nature of MS and the direct association between obesity (52) and functional disability in individuals (53).

## Relationship between glutamate and the incidence of anxiety and depression in multiple sclerosis

Many studies have focused on the role of cytokines, chemokines, and classical inflammatory mechanisms in the pathogenesis of MS (54). However, recent research has shown the importance of some inflammation-independent neurodegenerative mechanisms associated with mitochondrial dysfunction, iron deposition, and oxidative stress (55). In line with this, evidence also highlights the significance of neurotransmission systems in the onset and development of MS. For instance, it is known that an excess of glutamate is related to MS symptomatology. In fact, modulation of glutamate release and transport, as well as blocking its receptors, may be relevant targets for future therapeutic interventions (56). As commonly known, glutamate is the major excitatory neurotransmitter in the CNS and appears to play a central role in the communication between different brain cells; not only neurons but also oligodendrocytes, astrocytes, endothelial cells, and immune cells (57).

Glutamate excitotoxicity is a hypothesis that posits that excess glutamate leads to neuronal dysfunction and degeneration. In fact, glutamate excitotoxicity has been associated with various chronic neurodegenerative disorders (58). Specifically, in relation to MS, alterations in extracellular glutamate concentration, glutamate receptors, or the glutamate transporter could be associated with the disorder (55, 59), and these alterations could potentially explain the recently observed relationship between glutamate and functional disability in MS as a prognostic marker for the disease (60).

Additionally, excessive levels of glutamate appear to be directly related to the emotional aspects of dementia and psychiatric disorders. Metabotropic glutamate receptors (mGluRs) are attractive targets for therapies aimed at treating anxiety disorders (61). In various psychiatric disorders, glutamate has been associated with the perception of anxiety and depression (62). Similarly, this neurotransmitter has demonstrated its role in anxiety and depression in neurodegenerative disorders such as AD (63).

In fact, common mechanisms have been seen between depression and MS, including alteration of brain-derived neurotrophic factor, dysregulation of the hypothalamic–pituitary-thalamic axis, and inflammation or dysregulation of serotonin, norepinephrine, and glutamate (64). In relation to inflammation and glutamate dysregulation, a clear link has been established, as proinflammatory cytokines contribute to excitotoxicity in gray and white matter by impairing glutamate reuptake through astrocytes and oligodendrocytes, as well as monoaminergic neurotransmission in MS (65, 66). Specifically, IL-17A appears to be particularly relevant. In fact, a direct correlation has been observed between IL-17A and glutamate levels. IL-17A levels have also been associated with neutrophil expansion in CSF and disruption of the BBB, suggesting that Th17 cells may enhance and utilize glutamate excitotoxicity as an effector mechanism in the pathogenesis of MS (67).

Besides, these same authors further investigated the importance of IL-17A, observing its effect on the ability of astrocytes to metabolize and release glutamate. IL-17A may promote glutamate excitotoxicity by reducing the uptake capacity of astrocytes and converting glutamate into non-toxic glutamine, but also by stimulating Ca2 + −dependent glutamate release. Thus, the potential link between inflammation and neurodegeneration during the pathogenesis of MS becomes evident, identifying astrocytes as a potential target for achieving neuroprotective effects in the disease (68).

Interestingly, a relationship has also been seen between IL-1β and glutamate toxicity, with a decrease in glutamate toxicity when inflammation in the cerebral cortex is attenuated through elevated levels of IL-1β (69).

Ultimately, it is also noteworthy that by antagonizing the N-methyl-D-aspartate (NMDA) glutamate receptor, a rapid reduction in circulating levels of IL-6 is achieved, which in turn leads to a significant decrease in elevated levels of depression caused by glutamate over activity (70).

**Figure 1:**
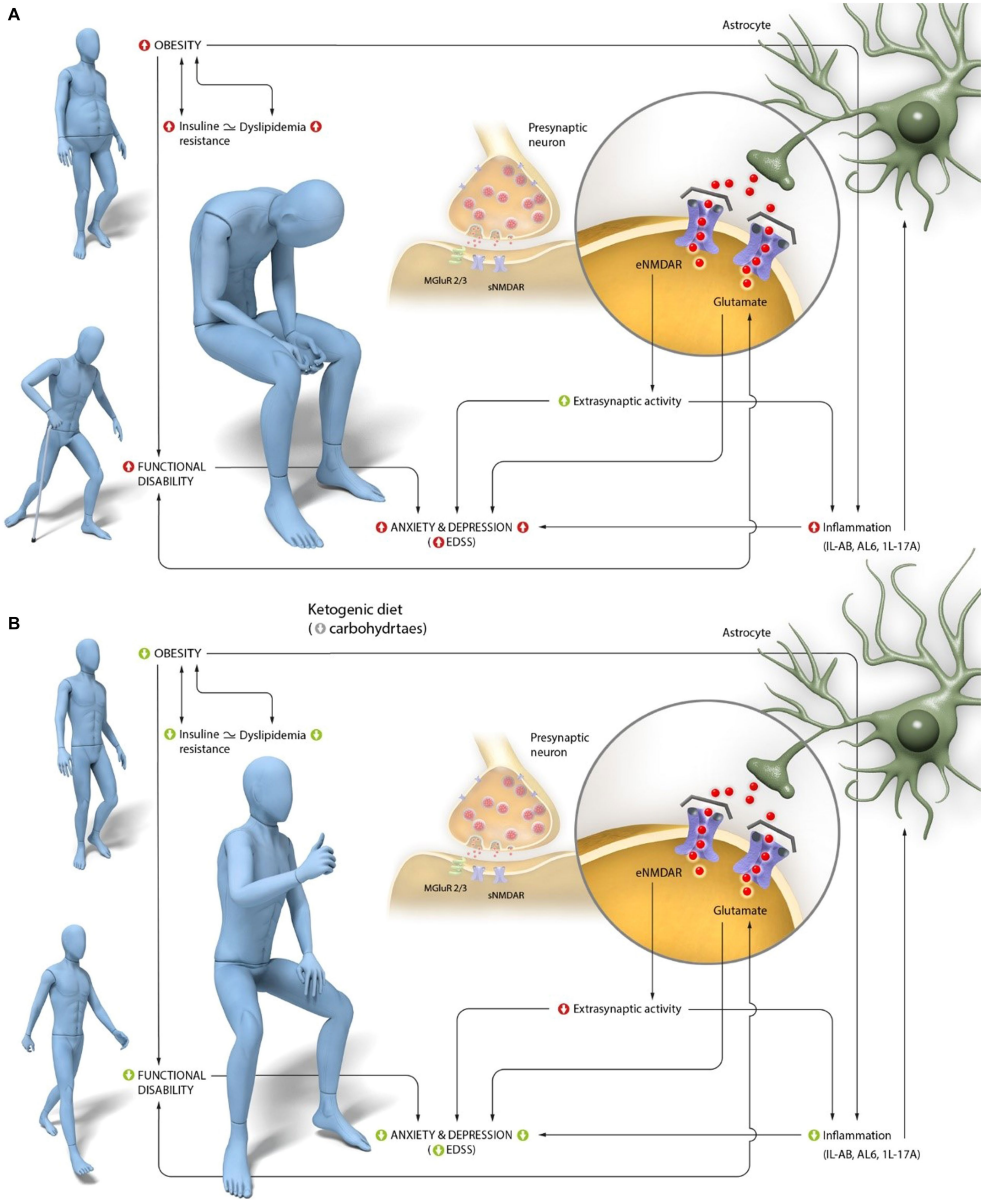
Interaction of central glutamate activity in anxiety and depression alterations, characteristic of Multiple Sclerosis (MS).**(A)** Peripheral and central pathogenic mechanisms in MS. Individuals with MS have a high prevalence of obesity, which is associated with insulin resistance. Obesity is directly linked to the characteristic functional disability of the disease and with increased central inflammation. This inflammation is primarily mediated in MS by an increase in IL-1β and its receptor (IL-1R), as well as an increase in IL-6, which stimulates T-cell activation and promotes IL-17A production, specifically related to functional disability. Disability, as well as inflammation in the CNS mediated primarily by these three interleukins, is associated with glutamate activity. Increased levels of glutamate are observed in areas of greater demyelination and axonal degeneration in MS. Finally, dysregulation of glutamate is associated with increased depression and anxiety, as the increased activity of IL-1β, IL-6, and IL-17A reduces glutamate uptake by astrocytes and stimulates its release at the extrasynaptic level. **(B)** Proposed mechanisms of action of a ketogenic diet (KD) in improving the perception of anxiety and depression in subjects with MS. The production of ketone bodies resulting from KD intake reduces obesity and improves insulin resistance, thereby enhancing functional capacity. This activity, along with the ability of ketone bodies to cross the BBB, may explain central glutamate activity, particularly at the extrasynaptic level, and through the reduction of IL-1β, IL-6, and IL-17A levels. Ultimately, these changes have an emotional impact, leading to a decrease in the perception of anxiety and depression characteristic of this pathology.

Therefore, considering all the mechanisms described and depicted in Figure 1A, antagonizing the action of this neurotransmitter could improve depression, among other symptoms, by reducing inflammation. However, regarding MS, there is not enough evidence to support the efficacy of memantine (an antagonist of AMPA glutamate receptors) in preventing cognitive decline or fatigue, despite memantine’s potential to reduce glutamate toxicity (71). However, the association between glutamate, emotional state, and neurodegenerative disorders is clear (72), highlighting the need to find alternatives to drugs in order to improve symptoms by modulating glutamate activity.

## Discussion

It is evident that MS is the result of a combination of genetic and certain environmental factors, including nutrition (73, 74). In this study, individuals with MS present a clear imbalance in the intake of macronutrients associated with obesity (especially abdominal obesity) and elevated levels of IL-6 (75). These imbalances are characteristic of the Western diet (high in energy, saturated fats, and sugars), which promotes inflammation associated with insulin resistance (76).

Ketogenic diet were initially designed to mimic the biochemical effects of intermittent fasting on the body (77). They involve restricting carbohydrate intake to a minimum while increasing the intake of fats, all while ensuring adequate protein intake (78).

The reduction in blood glucose, and consequent decrease in glycolysis, result in improved mitochondrial function by reducing the production of reactive oxygen species (79, 80). Additionally, there is an increase in blood ketone bodies, which, along with the low glucose availability, appears to have a neuroprotective effect by reducing inflammation (81, 82).

The first disease in which the effects of KD were studied was drug-resistant epilepsy (83). However, it was later observed that neurodegenerative diseases, such as amyotrophic lateral sclerosis, Alzheimer’s disease (AD), Parkinson’s disease (PD), Huntington’s disease, and MS share some pathogenic mechanisms and may also benefit from the effects of this diet (84). This is mainly because KD not only helps modulate mitochondrial function by reducing oxidative stress but also reduces neuroinflammation and promotes autophagy, regulates central and peripheral metabolism, and affects the intestinal microbiome in these diseases (85). All these mechanisms appear to have a particular relevance in MS, as KD have been shown to promote axonal remyelination (86). In fact, there is already clinical evidence of the benefits of this diet in people with MS. A randomized parallel-group three-arm pilot trial (NCT01538355) was conducted in RRMS subjects, where a slight reduction in EDSS scores was observed. Additionally, in a population sample of 65 individuals with relapsing MS, significant reductions in fat mass were observed after 6 months of treatment with a KD, leading to improvements in fatigue, depression, neurological disability, and inflammation (87). Moreover, it has been seen that following a KD for 6 months also significantly reduces serum neurofilament light chain (sNfL) levels, which are related to the impact on neuroaxonal damage in subjects with RRMS (88).

Finally, it is worth noting that, despite the study by Lee et al. (89), where the administration of a KD based on high amounts of medium-chain triglycerides (MCT) provided by coconut oil did not achieve a significant clinical improvement, positive changes in the development of the disease have also been seen following the intake of coconut oil (as a source of ketone bodies) combined with the administration of epigallocatechin gallate (EGCG). Specifically, it was seen that the intervention resulted in anthropometric improvements, characterized by a decrease in waist-to-hip ratio and body fat percentage, and an increase in muscle mass percentage. Additionally, the increase in paraoxonase 1, a marker associated with low levels of oxidative stress and inflammation, as well as serum albumin, also contributed to the reduction of cardiac risk in individuals with MS (90, 91). Furthermore, these changes were accompanied by improvements in functional capacity and anxiety (92).

Given the previously established relationship between functional disability and obesity, and the association of obesity with insulin resistance and dyslipidemia, functional improvement may be attributed to the regulation of these variables by ketone bodies. KD that promote these metabolites, when followed under the supervision of a healthcare professional, are a safe and effective treatment for weight loss in obese subjects, including those with comorbidities such as chronic kidney disease (93).

These diets are more effective in males than in females. However, due to potential hormonal influences in women, the effectiveness is the same for both sexes when reaching menopause (94). Thus, from an anthropometric perspective KD act by reducing visceral fat mass. On a serological level, they decrease fasting insulin levels by increasing insulin sensitivity (95), and regulate triglyceride levels, total cholesterol, glucose, and glycosylated hemoglobin (96). In fact, in animal models, it has been seen that a 3-day intervention with a KD leads to a decrease in fasting insulin levels, resulting in glucose intolerance, which may be associated with increased lipid oxidation (97). In this type of low-carbohydrate diet, it can be assumed that the excess lipids will be oxidized to provide energy in response to glucose scarcity, and the decrease in insulin levels is likely reversible and unrelated to any organic damage (97). The improvement in lipid profile is associated with a reduction in inflammation in obese individuals (98), with particular relevance placed on the decrease in interleukin IL-17, which is directly linked to the disease (99), as elevated levels of IL-17 are associated with disruptions in glucose and insulin metabolism (100).

Additionally, it should be noted that insulin resistance is common in MS and has been positively associated with functional disability determined by the EDSS (99, 101).

### Ketone bodies and their role in inflammation, depression, and anxiety in multiple sclerosis

Regarding the activity of ketone bodies in relation to inflammation, there is abundant scientific evidence supporting their role both peripherally and centrally (102–104). Furthermore, focusing on the key interleukins associated with the pathogenesis of MS, namely IL-6, IL-1β, and IL-17, the particularly active role of β-hydroxybutyrate (βHB) should be highlighted. This metabolite, capable of crossing the BBB, exerts protective effects against microglial activation, thereby reducing neuroinflammation and, specifically, IL-6 both *in vitro* and *in vivo* (105).

Its anti-inflammatory action also includes IL-1β activity modulation. βHB may suppress NLRP3 inflammasome activation and improve various inflammatory diseases. In fact, in human placental tissue cultures, treatment with βHB has been shown to suppress the secretion levels of inflammatory cytokines, such as interleukin IL-1β, IL-6, and IL-8. Additionally, βHB reduced the secretion of IL-1β and the amount of mature IL-1β protein induced by lipopolysaccharide (LPS) stimulation in the placenta. Furthermore, in the same study, it was also seen that βHB inhibited the secretion of IL-1β in human trophoblastic cells (106). It is important to recall that the direct involvement of the NLRP3 inflammasome in the onset and development of MS has been demonstrated in the experimental autoimmune encephalomyelitis (EAE) animal model, which justifies the particular relevance of IL-1β in MS (107).

Finally, it is interesting to mention that, when attempting to establish the mechanisms by which βHB determines the activation processes of BV2 microglial cells, it was observed that this metabolite had a neuroprotective effect on BV2 cells, significantly reducing the levels of expression of the proinflammatory interleukin IL-17 and increasing the levels of the anti-inflammatory interleukin IL-10. Thus, βHB appears to play a fundamental role in neuroprotection and the prevention of neurodegenerative diseases (108).

Individuals who consume unhealthy or Western-style diets rich in fat, sugar, and ultra-processed products are at a higher risk of depression and anxiety, with inflammation as the primary mechanism linking diet to these behavioral variables (109, 110). Specifically, one of the interleukins that stands out in this link is IL-6 in excess (111–115). In this context, KD in animal models have been shown to be effective in reducing depression, specifically through the restoration of microglial inflammatory activation and neuronal excitability (116). Furthermore, ketone bodies have shown to be efficient in reducing the perception of anxiety, as evidenced that in AD and in elderly humans the KD exhibited an anxiolytic effect (117, 118). Finally, it is significant to mention that this type of diet has shown to be particularly effective in reducing both depression and anxiety in PD (119, 120).

### Impact of ketone bodies on glutamate activity and the improvement of anxiety and depression in MS

Excessive extracellular concentrations of L-glutamate (L-Glu) can be neurotoxic and contribute to neurodegenerative processes in MS (121). L-Glu levels in CSF are lower in individuals with MS, correlating with functional disability (EDSS scale) in RRMS. Particularly relevant is also the relationship between L-Glu and levels of inflammatory molecules interleukin (IL)-2 or IL-6; thus, altered L-Glu is associated with disability progression and inflammation (122).

This inflammation can be promoted by obesity, since a clear link has been established between obesity and central inflammation, which in turn contributes to functional disability in MS. Thus, obese individuals with RRMS show greater clinical disability, as evidenced by elevated levels of proinflammatory molecules such as IL-6 and leptin in CSF, along with reduced concentrations of the anti-inflammatory cytokine IL-13. Interestingly, these subjects also show a positive correlation between CSF IL-6 concentrations and serum triglyceride levels, as well as the TC/HDL-C ratio, indicating that obesity and altered lipid profiles are associated with increased central inflammation and greater clinical disability in RRMS (19).

In line with this, a relationship has been established in individuals with AD between obesity and alterations in glutamate activity at the central level, specifically an increase in its binding to the extrasynaptic NMDA receptor. These alterations are mediated by insulin resistance linked to obesity, which in turn is associated with leptin resistance, ultimately contributing to the increased extrasynaptic activity of the neurotransmitter (123). It is interesting to note that this increase in extrasynaptic glutamate activity also explains the elevated levels of anxiety and depression in subjects with AD (63).

All these processes are shown in Figure 1B.

In summary, based on the analysis reported in this study, improving glutamate activity may be achieved by reducing obesity, which is associated with insulin resistance and dyslipidemia. These processes are further linked to a decrease in central inflammation, which is associated with dysfunctional extrasynaptic glutamate activity and emotional disturbances (124). Specifically, interleukins IL-1β, IL-6, and IL-17 appear to be particularly relevant, as they decrease following significant increases in blood βHB levels. Therefore, modulation of glutamate activity would imply an improvement in functional disability (60), associated with a reduction in the characteristic anxiety and depression experienced by these subjects. In the absence of effective pharmacological treatments, KD have shown to be particularly effective in achieving an anti-inflammatory effect through the modulation of glutamate activity. However, it is important to highlight that the administration of these diets must be guided by experts who monitor the individual’s response, as secondary issues such as constipation, nausea, vomiting, decreased appetite (125–127), and even transient hyperlipidemia (128), characterized by initial elevations in fasting serum total cholesterol, triglycerides, and low-density lipoproteins (LDL), have been reported (129).

## Author contributions

MPGP and JEdlRO: literature search and developed of the hypothesis. MC-B and CES-S: article reviews and wrote the manuscript. EN-I: writing-review. JMLR: editing. All authors contributed to the article and approved the submitted version.

## Funding

The present research was funded by the Catholic University Foundation “San Vicente Mártir”, for the research project. The “The Impact of Triglycerides on Multiple Sclerosis” (promotion code 2018-203-001).

## Conflict of interest

The authors declare that the research was conducted in the absence of any commercial or financial relationships that could be construed as a potential conflict of interest.

## Publisher’s note

All claims expressed in this article are solely those of the authors and do not necessarily represent those of their affiliated organizations, or those of the publisher, the editors and the reviewers. Any product that may be evaluated in this article, or claim that may be made by its manufacturer, is not guaranteed or endorsed by the publisher.
